# Development of an autonomous solvent extraction system to isolate astatine-211 from dissolved cyclotron bombarded bismuth targets

**DOI:** 10.1038/s41598-019-56272-7

**Published:** 2019-12-30

**Authors:** Matthew J. O’Hara, Anthony J. Krzysko, Donald K. Hamlin, Yawen Li, Eric F. Dorman, D. Scott Wilbur

**Affiliations:** 1 Nuclear Sciences Division, Pacific Northwest National Laboratory, 902 Battelle Blvd., PO Box 999, Richland, WA 99352 USA; 2 Department of Radiation Oncology, University of Washington, 616 N.E. Northlake Place, PO Box 355016, Seattle, WA 98105 USA

**Keywords:** Chemical engineering, Chemistry

## Abstract

Cyclotron-produced astatine-211 (^211^At) shows tremendous promise in targeted alpha therapy (TAT) applications due to its attractive half-life and its 100% α-emission from nearly simultaneous branched alpha decay. Astatine-211 is produced by alpha beam bombardment of naturally monoisotopic bismuth metal (^209^Bi) via the (*α, 2n*) reaction. In order to isolate the small mass of ^211^At (specific activity = 76 GBq·µg^−1^) from several grams of acid-dissolved Bi metal, a manual milliliter-scale solvent extraction process using diisopropyl ether (DIPE) is routinely performed at the University of Washington. As this process is complex and time consuming, we have developed a fluidic workstation that can perform the method autonomously. The workstation employs two pumps to concurrently deliver the aqueous and organic phases to a mixing tee and in-line phase mixer. The mixed phases are routed to a phase settling reservoir, where they gravity settle. Finally, each respective phase is withdrawn into its respective pump. However, development of a phase boundary sensor, placed in tandem with the phase settling reservoir, was necessary to communicate to the system when withdrawal of the denser aqueous phase was complete (i.e., the intersection of the two phases was located). The development and optimization of the autonomous solvent extraction system is described, and the ^211^At yields from several ~1.1 GBq-level ^211^At processing runs are reported.

## Introduction

Several alpha-emitting radionuclides are being considered for use in targeted alpha therapy (TAT)^1–5^. Of these, cyclotron-produced astatine-211 (^211^At, t_1/2_ = 7.214 h) shows tremendous promise in TAT applications due to its attractive half-life and its 100% α-emission from nearly simultaneous branched alpha decay (^211^At (5.87 MeV, 41.8%) and ^211^Po (7.45 MeV, 58.2%))^6^. Several clinical trials have been approved using ^211^At-labeled monoclonal antibodies. In former trials, chimeric antitenascin monoclonal antibody 81C6 treatment was administered within surgical resection cavities of malignant brain tumor patients^7^ and ^211^At-MX35 F(ab′)_2_ was administered into the peritoneal cavity for treatment of ovarian cancer^8^. At present, clinical trials are underway at the Fred Hutch/University of Washington Cancer Consortium^9^ using radioimmunotherapy with ^211^At for treatment of blood-borne cancers (myeloid leukemia, acute lymphoblastic leukemia, and myelodysplastic syndrome)^10^.

Astatine-211 is produced using alpha beam bombardment of naturally monoisotopic bismuth (^209^Bi) via the (*α, 2n*) reaction^11–13^. The Scanditronix MC-50 cyclotron at the University of Washington (UW) is capable of producing >4 GBq ^211^At with good radionuclidic purity using a 29.0 MeV alpha particle beam^11^. With sufficient production capacity in place, separation of ^211^At from the target material is one of the major challenges for routine processing.

The earliest ^211^At separations were performed by distillation from molten Bi targets^14–18^ or by solvent extraction of acid-dissolved targets^19,20^, frequently with diisopropyl ether (DIPE)^20–25^. The UW has refined a solvent extraction method that employs DIPE to isolate ^211^At from dissolved Bi targets; it has been comprehensively described by Balkin *et al*.^21^. The method is performed manually in a glove box. It involves (1) dissolution of the Bi metal target in ≥10 M HNO_3_; (2) evaporation of the resulting solution to dryness, leaving a Bi oxynitrate saltcake; (3) dissolution of the salts in 8 M HCl; (4) solvent extraction of ^211^At into DIPE; (5) followed by repeated washing of the DIPE with 8 M HCl; and (6) back-extraction of the Bi-isolated ^211^At into 4 M NaOH. The manual solvent extraction process is performed by mixing the two phases with a magnetic stir bar in a 20 mL glass LSC vial. At the conclusion of each step, the aqueous (dense) phase is withdrawn using a disposable transfer pipette; the phase boundary between the aqueous and organic liquids is identified visually. While the method provides reliable ^211^At yields (78 ± 11%) with high purity and labeling performance, its execution requires considerable skill by a trained radiochemist.

We have investigated techniques to adapt the manual solvent extraction process to a fluidically automated regime. In general, solvent extraction processes can involve an array of phase mixing techniques, including shaking and stirring (most frequently performed in the laboratory), and centrifugal contacting and mixer/settler operations (typically performed at the industrial scale)^26,27^. In contrast, applications involving small scale, in-line solvent extraction processes are relatively scarce in the literature. Rodríguez *et al*. have recently reviewed strategies for in-line automation of solid-phase and liquid-liquid (L/L) extractions in radioanalytical applications^28^. These involve L/L microextraction and dispersive L/L microextraction. In one medical radionuclide application, in-line solvent extraction of ^99m^Tc from dissolved, proton-bombarded ^100^Mo targets was performed using a stream of gas bubbles in a glass tube to mix the two liquid phases^29^.

In developing an automated solvent extraction system, the industrial-scale mixer/settler concept was adapted to an in-line, milliliter scale. Two pumps are used to deliver aqueous and organic phases concurrently into an in-line mixing device that induces a brief emulsification or a large/turbulent phase contact surface. We describe the performance of two in-line mixing devices: a glass bead-packed column and a serpentine (knotted tubing) mixer. Collection of the two mixed phases in a static phase settling reservoir on the other side of the mixing device allows the phases to gravity separate. The return of each phase into its respective pump is accomplished via the aspiration of the dense phase, followed by the light phase, from the phase settling reservoir. At this point, the organic phase (containing the extracted ion of interest) can be retained, while the post-contacted aqueous phase can be sent to waste. Then, the solvent extraction process can be resumed by performing the next step (e.g., organic phase washing with a fresh aliquot of acid).

A major challenge in automating a small-scale solvent extraction process is in accurately identifying the phase boundary, as will be discussed. Various optical methods have been previously employed to identify liquid phase boundaries, including the measurement of differences in refractive index, UV absorbance, and light reflection^30,31^. Given the dramatic differences in electrical conductivities between diisopropyl ether^32^ and the strongly acidic solutions required for Bi target dissolution and ^211^At solvent extraction^33,34^, we opted to develop a sensor that identifies the phase boundary by monitoring the phases’ ability to carry an electrical current.

Members of this research team have recently worked to develop fluidic systems to isolate ^89^Zr, a cyclotron-produced immunoPET radionuclide, from ^nat^Y metal foil targets^35–37^. More recently, efforts have been underway to improve the efficiency of the complex ^211^At isolation method performed at UW by integrating laboratory automation into the process. Previously, an in-line Bi target dissolution system was demonstrated to produce reproducible ^211^At release profiles using optimized nitric acid concentrations, flow rates, and volumes^38^. Herein, we describe the development of an autonomous in-line system for ^211^At isolation from dissolved and prepared Bi cyclotron targets via the acid/DIPE solvent extraction process. An in-line mixer/settler system was employed to briefly emulsify the two concurrently-injected phases and then allow the phases to gravity separate in a phase settling reservoir (PSR). A phase boundary sensor (PBS) was configured at the outlet of the PSR to provide real-time identification of the location of the unpredictable aqueous/organic interface. With these components in place, the extraction of ^211^At into DIPE, and subsequent repetitive acid washing of the DIPE, was possible. The development and characterization of the system, and its performance on ~1.1 GBq levels of ^211^At, are described.

## Results

### In-line mixer/settler system characterization

While manual solvent extraction in the laboratory is typically accomplished by shaking/settling or stirring/settling operations, these methods are generally not conducive to in-line approaches. We evaluated the performance of two in-line phase mixers (a ~1 cm^3^ glass bead-packed column mixer and a 1.2 m long tubular “serpentine mixer”) to affect the isolation of ^211^At from dissolved Bi-containing solutions. The results were compared to those achieved by UW’s manual (stirring/settling) solvent extraction process.

#### Bi^3+^ ion removal from DIPE during load/wash steps

We first evaluated the decontamination of Bi^3+^ ions from DIPE during the solvent extraction process. Solutions containing ~3 g dissolved Bi metal in conc. HNO_3_ were evaporated to dryness, leaving a white Bi oxynitrate residue of undefined composition. The residue was dissolved in 8 M HCl.

**Figure 1:**
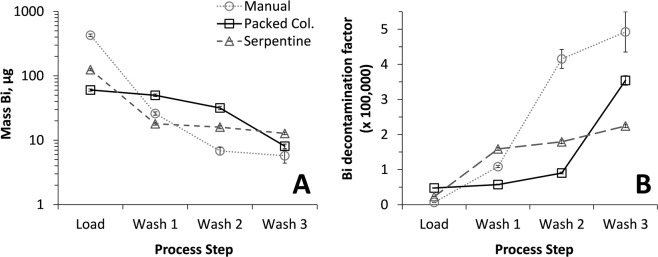
Comparison of Bi^3+^ contamination observed from manual solvent extraction method vs. in-line phase mixing methods. (**A**) Mass of Bi^3+^ determined in the DIPE phase following the initial sample load and after up to three wash cycles. (**B**) Calculated Bi^3+^ decontamination factors from an initial mass of ~3 g dissolved Bi metal in the load solution.

The resulting Bi-bearing solution was initially processed with the forward-extraction steps using DIPE in a magnetically mixed 20 mL vial, as per the manual wet-chemistry ^211^At isolation method (see Balkin *et al*.^21^ and the Electronic Supplementary Materials (ESM) Section). The concentration of Bi^3+^ remaining in the organic phase after the manual load and three successive organic phase wash intervals was determined by inductively coupled plasma – optical emission spectroscopy (ICP-OES). The mass of Bi (µg) measured in the organic phase is presented in Fig. 1A, and the calculated Bi decontamination factor (DF) at each stage in the manual solvent extraction process is presented in Fig. 1B (where DF is defined as the mass ratio of Bi dissolved in the originally-contacted acidic solution and Bi measured in the organic phase). The results indicate post-load Bi^3+^ contamination levels at 428 ± 19 µg. Following three successive washes with 8 M HCl, the Bi^3+^ contamination levels had dropped to 5.7 ± 1.3 µg.

Next, in-line mixing systems employing column and serpentine mixers were assembled and tested with a fluidic system to determine their effectiveness in Bi^3+^ ion removal from the organic phase. Results are provided in Fig. 1. It was observed that both in-line mixing methods resulted in Bi^3+^ contamination levels in the post-load organic phase that were considerably lower compared to the manual method (60 ± 2 µg and 125 ± 5 µg for the column and serpentine mixers, respectively). By the end of the triple-wash cycle, contamination levels had dropped to 8.1 ± 0.6 µg and 12.8 ± 0.5 µg, respectively. The three methods had approximately the same effectiveness at Bi^3+^ ion removal across the load/wash sequence (each ending with <13 µg Bi); the manual solvent extraction method resulted in the highest Bi DF (~4.9 × 10^5^), followed by the column and serpentine mixers (~3.5 × 10^5^ and ~2.2 × 10^5^, respectively).

#### ^211^At solvent extraction during load/wash steps

Each of the two in-line mixing methods were subsequently evaluated for their effectiveness at ^211^At extraction into DIPE. A surrogate dissolved target solution was prepared by spiking freshly isolated ^211^At (~1.9 MBq) into freshly-dissolved Bi metal pieces (using 10 M HNO_3_). The solution was evaporated to dryness, and the salts were dissolved in 8 M HCl (*vide supra*). A pair of syringe pumps was used to deliver the ^211^At-/Bi-bearing solution and DIPE concurrently through a mixing tee and in-line mixer. At the conclusion of the delivery, the phases were allowed to gravity separate, and then a small volume of the DIPE solution (10 µL) was sampled and analyzed for ^211^At activity levels. Next, each phase-separated liquid was withdrawn into its respective syringe pump, and the phase mixing→phase settling→DIPE sampling process was repeated three more times. We observed that ^211^At extraction yields were nearly quantitative after the first pass, as shown in Table 1; the column and serpentine mixers provided extraction yields that were not statistically distinguishable from each other.

**Table 1:** Solvent extraction yields of ^211^At spikes from Bi^3+^-loaded solution into DIPE as a function of the number of cycles delivered through the column and serpentine mixers^a^.

Cycle	Column mixer, %	Serpentine mixer, %
1	95.4 ± 4.8	98.7 ± 4.9
2	94.8 ± 4.7	100.5 ± 5.0
3	94.1 ± 4.7	97.3 ± 4.9
4	94.3 ± 4.7	102.9 ± 5.1

^a^Experimental uncertainty was ± 5% at 1σ, as determined by the reproducibility of pipetting 10 µL of DIPE.

After confirming that both of the in-line mixers could effectively transfer ^211^At into the organic phase during a load sequence, the complete forward extraction load/wash process was evaluated for the manual method and the two in-line mixing assemblies. Surrogate dissolved targets were prepared with spikes of ^211^At (~1.9 MBq) added to Bi metal (~3 g), then dissolved and prepared as described above. Following each DIPE solution load and sequential wash step, a small volume of the aqueous phase was sampled and analyzed for ^211^At activity concentration. This activity was corrected for aqueous phase volume and compared to the known activity initially present in the prepared surrogate target solution. The performance of each method was evaluated in triplicate.

The results in Table 2 present the calculated ^211^At activity fraction remaining in the aqueous phase following each load and wash step (see Experimental Section and the ESM for details on load and wash step volumes for the in-line and manual methods, respectively). The manual method provided somewhat variable ^211^At extraction during load, with 10.5 ± 5.8% of ^211^At remaining in the aqueous phase. The subsequent 8 M HCl washes of the ^211^At-loaded DIPE resulted in minor and slightly increasing ^211^At losses at each stage. Overall, the manual method resulted in an extraction of all but 15.0 ± 5.9% of the ^211^At activity originally present.

**Table 2:** ^211^At solvent extraction performance comparison of the manual vs. the two in-line mixing methods. Values represent the percentage of ^211^At measured in the aqueous phase at the conclusion of each phasemixing step^a^.

Step	Manual method		Column		Serpentine	
	Loss	±1s	Loss	±1s	Loss	±1s
Load ^b, c^	10.5	5.8	4.3	0.5	5.8	1.7
Wash 1	1.2	0.4	0.9	0.2	1.2	0.6
Wash 2	1.4	0.3	1.2	0.3	1.5	0.4
Wash 3	1.9	0.6	1.2	0.3	2.0	0.1
**Cumulative loss**	**15.0**	**5.9**	**7.5**	**0.7**	**10.6**	**1.9**

^a^Each method was performed in triplicate.

^b^~3 g dissolved Bi metal spiked with ^211^At, evaporated to dry salts, and dissolved in 8 M HCl.

^c^For the in-line mixing methods, the load solution was delivered 2x through the mixers.

The in-line mixing methods were evaluated next. Surrogate ^211^At-bearing dissolved target solution was delivered concurrently with DIPE two times through the in-line mixers prior to sampling for ^211^At activity determination. Passage of the aqueous and organic phases through the column and serpentine mixers during the dual load steps succeeded at extraction of all but 4.3 ± 0.5% and 5.8 ± 1.7% of the ^211^At, respectively. As with the manual method, each successive organic phase wash with 8 M HCl resulted in a small, incrementally increasing activity loss into the aqueous phase. Cumulative ^211^At losses for both in-line methods were below ~11%; they were within experimental uncertainty at ±2 s. However, given the column mixer’s slightly better performance relative to the serpentine mixer, it was selected to be integrated into the engineered fluidic system.

### Phase boundary sensor (PBS) characterization

The development and integration of an aqueous/organic PBS into the fluidic system would make it possible for the apparatus to trigger an event when the aqueous/organic phase boundary is identified. In this manner, the unpredictable volumes of the aqueous and organic phases in the PSR could be accommodated. The system aspirates the (dense) aqueous phase into the aqueous solution handling pump until the phase boundary is sensed (i.e., all aqueous solution has been withdrawn from the PSR). At this point, the aqueous solution handling pump ceases aspirating, and the organic phase handling pump aspirates the remaining organic phase from the PSR.

The PBS was designed to trigger an event based on a change in the electrical resistivity measured when a highly conductive aqueous solution (acid) passed through the monitored flow channel, followed by a poorly conductive organic liquid (DIPE). The resistivity measured across two electrical leads spikes to a “high” state when the organic solution enters the zone between the two leads. Below, the behavior of the PBS in acid/organic solutions is reported.

Given its well-established electrical conductivity and chemical resilience to strong acids, Pt wire was chosen as an electrode material to be tested in the PBS for the acid/organic phases. Using a data-logging potentiostat set up in open circuit potential (OCP) mode, we evaluated the Pt wire sensor performance as a function of time while it was repeatedly exposed to strong HCl and acid-contacted DIPE. In OCP mode, the potentiostat applies no current or voltage; it simply monitors the voltage across a circuit (in our case, the Pt wires positioned within the PBS flow channel). Using the potentiostat in this manner, the integrated switching logic of the digital input signal on our solvent extraction platform was monitored (i.e., input voltage level).

**Figure 2:**
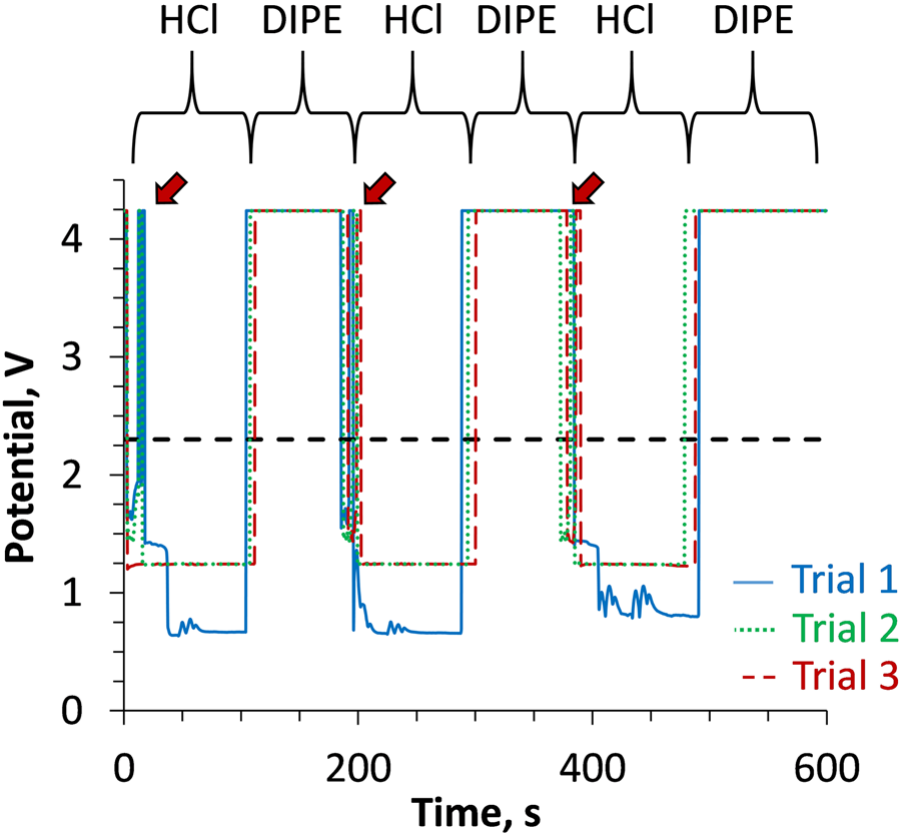
Voltage potentials measured across the Pt coil electrode terminals connected to the input logic board during alternating deliveries of 8 M HCl and DIPE solutions. Horizontal dashed line indicates approximate potential above which the sensor input logic is switched from “low” to “high” state. Arrows indicate a region of brief sensor instability upon exposure to acid.

A testing program was set up wherein a syringe pump was programmed to aspirate liquid from a phase-separated reservoir of 8 M HCl and DIPE while logging the voltage levels across the Pt leads via the potentiostat. Each trial consisted of three withdrawals of HCl/DIPE phases from the PSR; data traces across three trials were recorded. The potentiostat traces are shown in Fig. 2.

When the Pt coil leads were exposed to acid, a low-resistivity condition was identified; the OCP recorded a low potential across the input logic circuit. When the input signal was below ~2.3 V potential (dashed line), the input circuit remained in the “low” state.

When the sensor signal rose above the ~2.3 V threshold potential, it switched to the “high” state. The high voltage is associated with a high resistivity between the Pt coil leads. Since DIPE is a vastly poorer electrical conductor than strong acid (*vide supra*), the resistivity across the sensor would be expected to increase significantly once the organic solution passed between the Pt coil electrodes within the monitored flow channel.

The data traces in Fig. 2 show that there is a brief period of sensor instability that occurs within a few seconds after 8 M HCl is introduced to the sensor (rapid fluctuation of signal above and below the sensor switching voltage). In order to assure that a premature switching event would not occur during aspiration of acid through the PBS, the software program was modified so that the PBS would exclude input data during this brief period of instability immediately following acid addition (3 s). Conversely, the voltage shift observed at the acid/DIPE interface did not exhibit the same instability; once the DIPE was in contact with the sensor, the voltage immediately went “high”, assuring that the switching mechanism would be promptly activated.

### Fluidic system performance testing on cyclotron bombarded Bi targets

After evaluations of in-line solvent mixing and phase boundary sensing were completed, a fully autonomous solvent extraction system capable of emulating the manual wet chemical ^211^At isolation process was designed, constructed, and programmed. The programming of the system was initially refined while running non-radiological solutions, and then further refined on ^211^At-spiked solutions in the UW glovebox. Ultimately, a series of nine performance tests were made on freshly-bombarded Bi cyclotron targets that contained 1.07 ± 0.02 GBq of ^211^At (at end-of-bombardment (EOB)). The targets used in this series contained 4.8 ± 0.5 g of Bi metal.

For each GBq-level run, the Bi targets were dissolved using the automated target dissolution station that was previously described^38^. The dissolved Bi effluents were routed from the dissolution block to a heated distillation chamber that brought the solutions to dry Bi oxynitrate salts. After the salts were cooled, the fluidic system’s pump 1 injected 8 M HCl onto the salt residue; a combination of magnetic mixing and aspiration/dispensation of the HCl solution (pump 1) resulted in complete dissolution of the salts after several cycles had been performed. The prepared target solutions were then ready for in-line solvent extraction.

**Figure 3:**
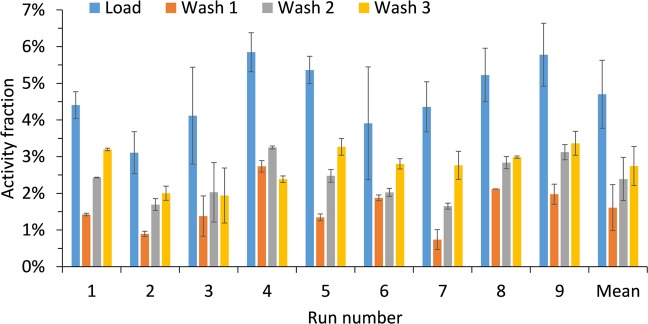
Observed ^211^At losses in the aqueous phase following the prepared target/DIPE load step (4.7 ± 0.9% loss) and three successive washes with 8 M HCl (1.6 ± 0.6%, 2.4 ± 0.6%, and 2.7 ± 0.5% loss, respectively).

The loss of ^211^At to the aqueous phase was tracked at each stage of the processing runs (load and three successive washes). Each load and wash fraction was collected separately and analyzed after completion of the runs. The data presented in Fig. 3 shows the incremental ^211^At loss at each stage of the forward extraction method for each target.

The data shows that the majority of the ^211^At loss occurred during the prepared target/DIPE mixing (“Load”) step. The average percentage of ^211^At remaining in the aqueous phase following the Load step was 4.7 ± 0.9%. These results compare favorably to the manual processing runs, wherein the ^211^At found in the post-contacted load solution was 10.5 ± 5.8% (Table 2).

After ^211^At loading, some of the DIPE-extracted ^211^At was incrementally lost to the three successive DIPE washes with 8 M HCl. The degree of ^211^At loss increased slightly with each wash, resulting in 1.6 ± 0.6%, 2.4 ± 0.6%, and 2.7 ± 0.5% lost in washes 1 through 3, respectively. These incremental ^211^At losses were likewise observed in our earlier trials when comparing the manual and in-line mixing methods (Table 2).

Overall, the total ^211^At loss to the aqueous phase was 11.4 ± 1.3% across the study. This was the fraction of ^211^At activity that was not retained in the organic phase (routed to waste) during the pump 1/2 forward extraction operations that involved the packed column-based mixer/settler system. The cumulative ^211^At recovery (obtained after target dissolution → target solution evaporation → recovered activity from the dissolved Bi salts → the above-described solvent extraction load/wash steps) was calculated to be 79.4 ± 4.5%.

## Discussion

### Necessity of the PBS

Development of a procedure that allows “automatic” solvent extraction in the isolation of ^211^At is difficult, if not impossible, given the unpredictable nature of the aqueous/organic phase volume changes encountered during the target preparation and solvent extraction processes. Although the DIPE and 8 M HCl reagents used in the method were pre-contacted with each other prior to use (to minimize volume change during execution of the method), the precise volume of dissolved target/HCl/DIPE in each phase was not known. In the manual method, these volume inconsistencies are inconsequential since phase separation at each step is accomplished by pro-active (visual) means.

Due to tolerances in production of the Al backing for the cyclotron targets, and machining the Bi metal melted onto the Al backing, the Bi metal targets used for ^211^At production had varying mass. In our initial studies, the Bi metal in the target assemblies ranged between 3.5 and 6.5 g. Later changes in machining tolerances, and adapting a weight-based removal of excess Bi from targets, reduced the Bi mass uncertainty substantially to 4.8 ± 0.5 g for targets employed in the GBq-level study. Because Bi metal mass in the target assemblies was determined by weight difference pre- and post-dissolution, the quantity of Bi metal could not be known in advance. Thus, dissolution of the Bi metal in nitric acid and subsequent heating/distillation of the acid resulted in unknown quantities of Bi-bearing salts. Further, differences in salt quantities affected the level of dryness obtained for a given thermal treatment interval, which in turn affected the chemical composition (e.g., bismuth:nitrate ratio and level of hydration) of the Bi oxynitrate salt^39–42^.

These unpredictable salt quantities and compositions resulted in unpredictable solution volumes when the salts were dissolved with 8 M HCl in preparation for ^211^At solvent extraction. The HCl-dissolved target solution volumes varied as a function of starting Bi metal mass and the HCl volume added (see data presented in the ESM).

Additionally, during the initial contact of the dissolved target and DIPE solutions, some volume change between the phases was observed, the degree of which was generally driven by the ionic strength of the dissolved target solution. Phase volume changes during the three DIPE solution wash steps were nominal, since the 8 M HCl solution used in the washes was equilibrated with DIPE prior to introducing the reagent into the fluidic system.

**Figure 4:**
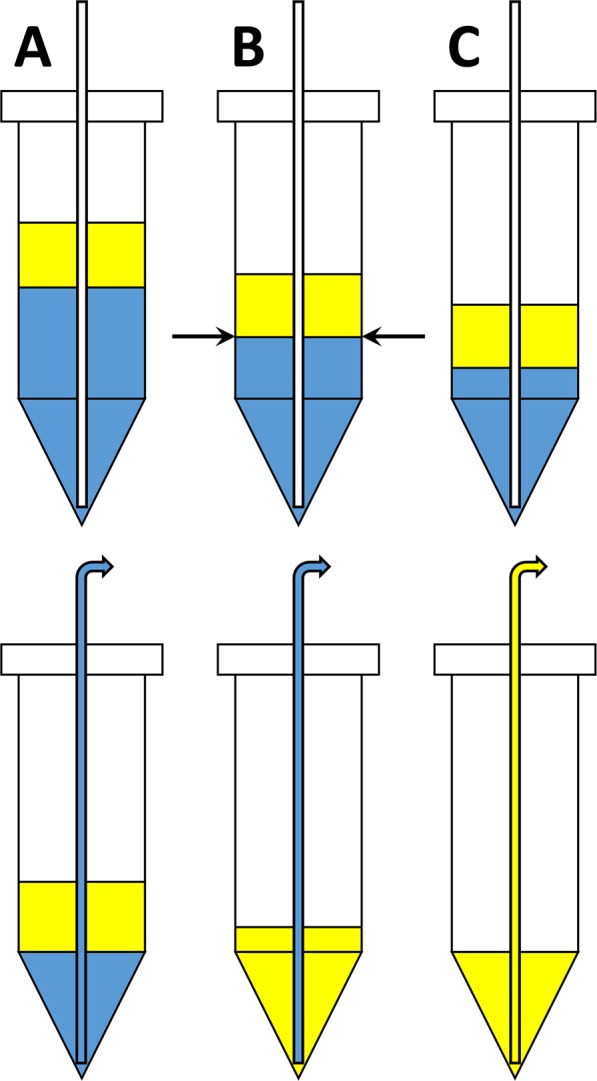
(Top) Illustration of the effects of “high” (**A**), “normal” (**B**), and “low” (**C**) mass Bi targets during automatic solvent extraction. Arrows indicate the liquid phase boundary based on a predictable, “normal” condition. (Bottom) Effects of the aspiration of a fixed aqueous phase volume based on a “normal” target.

The general issues with “automatic” solvent extraction from unknown input volumes are illustrated in Fig. 4. The top row shows three hypothetical conditions encountered upon delivery of three dissolved Bi targets and a fixed organic solution volume to a PSR. “Heavy” (A), “normal” (B), and “light” (C) Bi targets result in decreasing aqueous phase volumes, respectively.

The bottom row of Fig. 4 illustrates the effect of a fixed aqueous phase aspiration based on a pre-programmed “normal” target mass. In condition (B), the aspiration volume is sufficient to remove all of the dissolved target solution, thus enabling effective phase separation prior to initiation of the organic phase wash cycles. In condition (A), the same aspiration volume is insufficient to remove all of the dissolved target solution. This results in excess acid/Bi salt residing in the reservoir at the conclusion of the volume withdrawal. As a consequence, the subsequent wash steps with clean 8 M HCl are ineffective at removing Bi from the organic phase, since the remaining Bi-bearing target solution will simply be diluted at each wash step. In condition (C), the aspiration volume is excessive; all of the aqueous phase is withdrawn, along with a portion of the organic phase. Under this condition, some of the ^211^At-bearing organic phase is sent to waste along with the post-contacted dissolved target solution. The ^211^At yields will consequently suffer.

Given the unpredictable aqueous solution volumes going into the front end of the solvent extraction system, the process could not be achieved by performing a fixed sequence of pre-programmed (“automatic”) commands. Our response to this issue was to develop a sensor that could identify the phase boundary between the two immiscible liquids. With such a sensor, a fully “autonomous”, multi-step solvent extraction process became possible.

It is worth noting that the presented phase boundary sensing approach can conceivably result in the separation of arbitrary amounts of different aqueous/organic liquid phases from one another, so long as the liquid interface is well defined and the electrical conductivity of each liquid is sufficiently different.

### ^211^At yields obtained from the autonomous fluidic system

The solvent extraction steps encompassing the loading of ^211^At into the organic phase from the prepared target solution and the triple wash steps to remove Bi^3+^ ions from the organic phase resulted in 88.6 ± 1.3% ^211^At recovery in the DIPE. This was based on careful accounting of the ^211^At present in the load and triple wash solutions (11.4 ± 1.3% ^211^At loss in aqueous phase, Fig. 3).

When considering incremental ^211^At losses in the steps leading up to the conclusion of the solvent extraction process, the cumulative ^211^At yield was 79.4 ± 4.5%. This additional ~9.2% loss was attributed to several possible sources: (1) the inability to recover all ^211^At from the aluminum target assembly that backs the Bi metal layer; (2) ^211^At volatilization during the evaporation of HNO_3_ during the distillation step; (3) spatter of Bi oxynitrate salts on the upper walls of the distillation chamber caused by the boiling HNO_3_ during distillation; and (4) ^211^At-bearing residues remaining in the distillation chamber following aspiration of the HCl-dissolved Bi-bearing salts. A direct comparison of the ^211^At yields up to this point in the manual process is not possible, as Balkin *et al*.^21^ only reported mean yields in the final ^211^At product fraction (which included back-extraction of ^211^At into 4 M NaOH). However, our yields at the conclusion of the acid → DIPE cycles (79.4 ± 4.5%, n = 9) compare favorably with those reported by Balkin *et al*. for the entire ^211^At isolation process (78 ± 11%, n = 55). Further, the uncertainties associated with the autonomous vs. manual methods indicate that the presented method may be capable of providing more reproducible ^211^At yields (although the number of trials was fewer in the current evaluation).

### Fluidic system expansion

The autonomous acid/DIPE solvent extraction system described herein was further expanded to include the capability to perform an autonomous DIPE/4 M NaOH back-extraction step, during which the isolated ^211^At in DIPE is transferred back to an aqueous phase. This back-extraction module allowed for execution of the complete, end-to-end solvent extraction process described by Balkin *et al*.^21^. The expanded system was comprised of three syringe pumps (pump 3 was for handling the NaOH-based back extractant) and a separate in-line mixer, PSR, and PBS. The PBS for DIPE/base phase identification employed the same electrical conductivity monitoring approach, although Pt electrodes were not practical and a new electrode material needed to be identified. Ultimately, the system dispensed the isolated ^211^At product in a small volume of 4 M NaOH. A description of the back-extraction module development and its performance in the end-to-end process with clinical levels of ^211^At will be described in a future article.

## Methods

### Reagents

Hydrochloric acid (HCl) and nitric acid (HNO_3_) were ACS Certified grade or higher (Fisher Scientific, Waltham, MA). Dilutions of these reagents were prepared from deionized water (≥18 MΩ∙cm) using a Barnstead Nanopure Diamond water purification system (Dubuque, IA). Scintillation cocktail was Ultima Gold™ AB (PerkinElmer, Waltham, MA).

Diisopropyl ether (DIPE) was Certified ACS grade (Fisher Scientific). For method development, DIPE was used as-is; for high-level (e.g., GBq-level) ^211^At isolation runs, DIPE was distilled prior to use to assure removal of reagent stabilizer (e.g., butylated hydroxytoluene (BHT) or hydroquinone).

Bi pellets of 99.999% purity, used in the manufacture of Bi target assemblies^11^, were acquired from Alfa Aesar (Ward Hill, MA). Simulated dissolved Bi metal targets employed Bi metal pieces of the same purity (Sigma-Aldrich, St. Louis, MO). Platinum wire was 0.25 mm dia. and had a metal purity of 99.9% (Alfa Aesar). Coiled Pt electrodes were prepared by wrapping the wire around an 18 gauge hypodermic needle. The electrodes were secured and sealed into the PBS housing with ferruled 1/4-28 fittings.

### ^211^At production

The Scanditronix MC-50 cyclotron at the University of Washington Medical Cyclotron Facility (UWMCF) routinely produces ^211^At via the ^209^Bi(*α, 2n*)^211^At reaction. It is capable of producing external alpha (He^2+^) beams in the energy range of 27.0–47.3 MeV. Gagnon *et al*. have described the design, preparation, and performance of the Bi targets^11^. For the nine Bi metal targets bombarded for the final fluidic system performance evaluation, the cyclotron ran an integrated target current of 38 µA·h, which required 0.75–1.2 h of beam time to produce 1.07 ± 0.02 GBq (28.8 ± 0.5 mCi) of ^211^At (EOB).

### Manual method

The routine manual method for ^211^At isolation from solution-prepared cyclotron bombarded Bi metal targets by solvent extraction has been described by Balkin *et al*.^21^. A brief summary of the manual method (including target preparation and solvent extraction steps) is presented in the ESM.

### Fluidic system components

Fluids are delivered using two 48,000 step digital syringe pumps (model V6, Norgren, Las Vegas, NV) coupled to 8-position distribution valves at their heads (Norgren). Pumps 1 and 2 are configured with 25 and 10 mL volume displacement (“zero dead volume, ZDV) syringes, respectively (Flex Fluidics, Las Vegas, NV). The dual pumps are assembled into a metal box provided by J-Kem Scientific (Model 2200, St. Louis, MO). Accessible from the rear of the box is the pump’s digital input/output board ports. The pumps’ distribution valves are plumbed using 0.75 mm ID × 1/16″ OD Teflon^®^ FEP tubing. The tubing is connected between the distribution valves and the reagent reservoirs, waste receptacles, and phase mixing/settling hardware using polyether ether ketone (PEEK) or ethylene tetrafluoroethylene (Tefzel^®^, ETFE) ¼−28 flangeless nuts with Tefzel ferrules (Upchurch Scientific, Oak Harbor, WA). The fluidic system is controlled by a laptop PC using a PNNL-modified version of *KemPump* software (JKem Scientific).

### In-line mixer/settler systems

**Figure 5:**
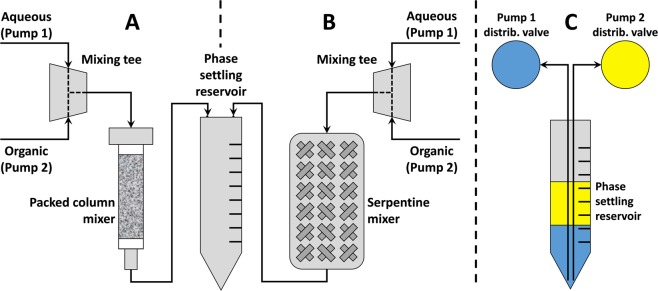
Schematic of a column (**A**) and serpentine (**B**) mixer positioned between a mixing tee and a conical vial used as a phase settling reservoir (PSR). Pump 1 delivers acidic solution, and pump 2 delivers organic liquid (DIPE) simultaneously into the mixing tee and in-line mixer. Following phase separation, the liquids are sequentially aspirated back into their respective pump (**C**).

The two liquid phases are delivered simultaneously from their respective syringe pump: aqueous solution from pump 1 and organic liquid from pump 2. The liquids intersect at a mixing tee and then pass into a phase mixer – a tortuous path through which the fluids are intimately co-mingled. Beyond the phase mixer is a PSR – a container used to collect the mixed phases and provide a static condition in which the phases can gravity separate (Fig. 5).

The first phase mixer evaluated was a 1 cm^3^ SPE column (Sigma-Aldrich) packed with 212 µm dia. silanized glass beads (Sigma-Aldrich) (Fig. 5A). It was fitted with a custom-machined cap at the inlet end that enabled fluids to be delivered to the column via with a male luer adapter. Additionally, we evaluated a Super Serpentine Reactor™ (GlobalFIA, Fox Island, WA), which was a 1.2 m length of 0.75 mm ID/1/16″ OD Teflon FEP tubing (0.53 mL internal volume) braided tightly through a perforated plate (Fig. 5B).

The flow rates of each pump were programmed so that the total volume of each respective phase was delivered towards the mixing tee over an equal time period. Hence, the syringe volume:dispensation flow rate ratio was the same between aqueous and organic phase syringes: typically 1.25 min per full syringe stroke (20 mL∙min^−1^ for a 25 mL syringe; 8 mL∙min^−1^ for a 10 mL syringe). The two solutions were merged at the mixing tee, and were then passed through the in-line mixer. A quick dispensation of air (at same flow rates as above) assured that the fluids were “chased” through the apparatus at the conclusion of the syringe stroke.

The tortuous path of the serpentine mixer causes in-line mixing to occur^43–45^. For the column mixer, the two liquids were delivered to the column of glass beads, thus creating intensively mixed phases as they were driven through the bed of small spheres. Upon exiting either the serpentine or column mixer, the biphasic mixture was delivered to a PSR (centrifuge tube or syringe barrel), where the two phases quickly separated. The phase settling interval was 30 s, which was ample time for the organic/aqueous phases to separate and for most of the fine solution-entrained bubbles from the air push to rise to the surface of the DIPE.

Next, tubing that connected each syringe pump’s distribution valve to the bottom of the reservoir was used to withdraw first the (dense) aqueous phase, and then the organic phase, back into each respective pump (Fig. 5C). In this manner, the processing cycle was set to be repeated. Alternatively, the aqueous phase could be dispensed to waste, the syringe pump rinsed with clean 8 M HCl, and re-loaded with 8 M HCl rinse solution prior to the next phase mixing interval.

### Phase boundary sensor (PBS)

**Figure 6:**
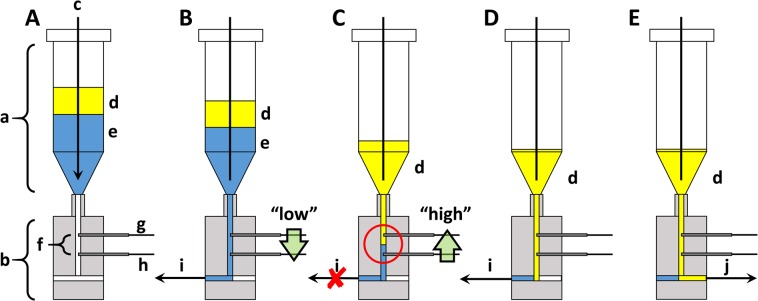
Sequence of steps in the automated biphasic liquid separation system. (**A**) Introduction of biphasic solution into the PSR from an in-line phase mixer and allowing phases to separate; (**B**) Withdrawal of aqueous phase to aqueous pump, PBS is in the “low” state; (**C**) Triggering of the PBS to the “high” state, and ceasing the PBS monitoring cycle; (**D**) Withdrawal of the remaining aqueous phase in the common fluid channel; (**E)** Withdrawal of organic phase to organic pump. Label descriptions are provided in Table 3.

The digital syringe pumps employed in the described fluidic processes are equipped with an external input signal processor board that allows voltage (0–5 V) to be monitored in real time; we took advantage of this feature to implement a PBS. The PBS body, which is machined out of a Teflon cylinder, is mounted to the base of a PSR (20 mL syringe barrel). The outlet of the PSR is connected to the inlet of the PBS with a luer/¼-28 coupler. Near the bottom of the PSR is a fluid channel in a “tee” configuration, which allows fluids to be withdrawn from the reservoir by either pump 1 or pump 2. Two electrodes project into the fluid channel, each held in place by ferruled ¼–28 fittings; they are positioned 2 cm apart. The electrodes are connected to the +5 V and ground terminals of the pump’s input signal processor. Aqueous and organic liquids are simultaneously passed through a mixing tee and phase mixer from pump 1 and pump 2. Upon exiting the phase mixer, they are collected in a PSR perched atop the PBS (Fig. 6A).

The fluid handling software was programmed to read the signals from a simple logic gate binary system offered by the pump’s signal input board. After withdrawing 25 µL of aqueous solution through the PBS (at 40 mL∙min^−1^ flow rate), the input board’s signal is momentarily read. If the resistivity of the solution is small, then perform task 1; if the resistivity is large, then perform task 2. Task 1 instructs pump 1 to continue withdrawing solution through the sensor (at 25 µL increments), since acid is present between the two electrodes within the PBS’s flow channel (sensor reads “low”, Fig. 6B). Task 2 instructs the pump to cease withdrawing solution through the sensor once the organic phase has entered the sensor (e.g., the phase boundary has been detected, sensor reads “high” (Fig. 6C)). Once the phase boundary is sensed, pump 1 aspirates a small volume of the aqueous solution to clear the dead volume between the Pt electrodes and the tee at the base of the PBS (Fig. 6D). Finally, pump 2 withdraws the isolated organic phase from the PSR (Fig. 6E).

### Engineered solvent extraction system and sample processing

**Figure 7:**
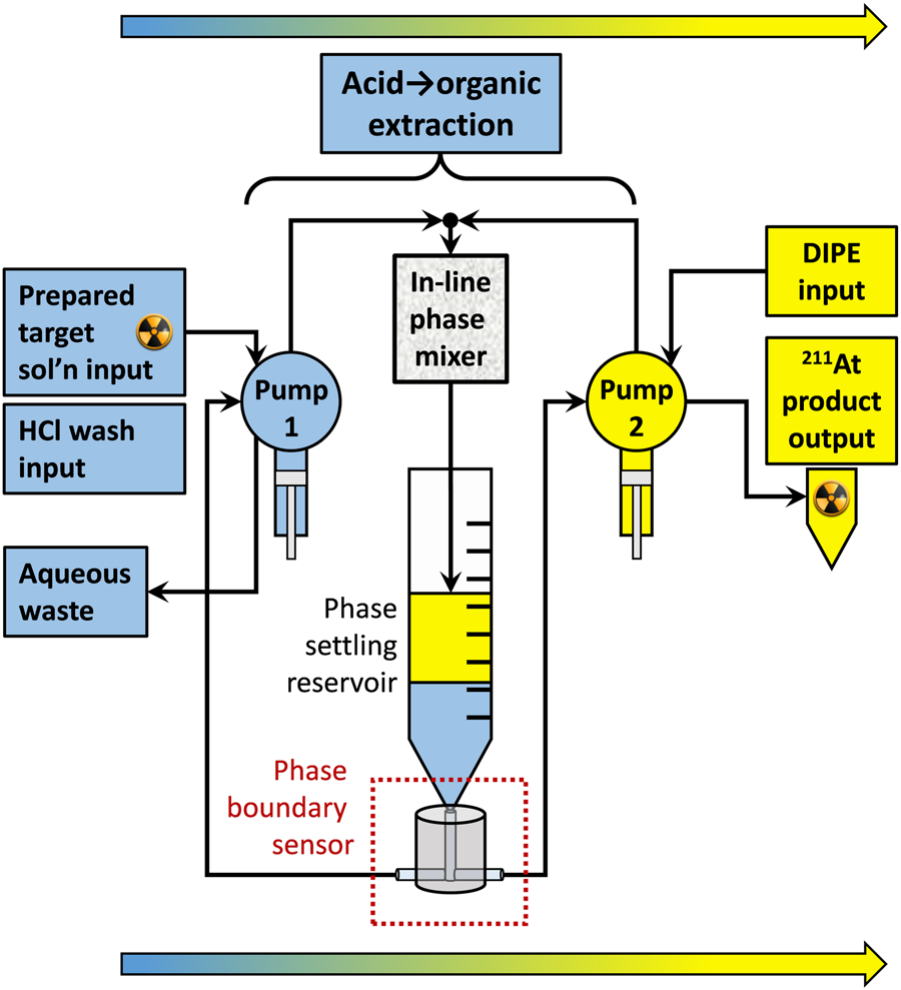
Schematic of the dual-pump fluidic system used to perform autonomous solvent extraction for the isolation of ^211^At from dissolved Bi target solutions.

The fully engineered autonomous solvent extraction system is comprised of two digital syringe pumps, a forward extraction mixer/settler system (1 cm^3^ column mixer), and a PBS (Fig. 7). The first pump handles the prepared target solution and acidic wash solutions; the second pump handles the DIPE solution. The syringe pumps were inverted, with the distribution valves below the syringe (note that Fig. 7 is not illustrated in this configuration). In this manner, liquid phases could be efficiently “chased” with air after each liquid delivery stroke.

The system was designed to have the syringe pumps and all peripheral components of the mixer/settler arrangement organized in a small space immediately in front of the pumps. In this compact configuration, the tubing connections are kept as short as possible, and they are less likely to be damaged by the cumbersome glovebox gloves that are necessary to be used when ^211^At processing is underway. An image of the fluidic system is presented in the ESM.

The sequence of high-level steps performed by the fluidic system is described in Table 4, and the delivered reagents and volumes at each step in the process is presented in Table 5.

**Table 3:** Identification of labels in Fig. 6.

Label	Description
a	Phase settling reservoir (PSR)
b	Phase boundary sensor (PBS)
c	Biphasic influent from phase mixer
d	Organic phase
e	Aqueous phase
f	Electrodes penetrating into fluid pathway
g	Electrode to +5 V input terminal
h	Electrode to ground terminal
i	Aspiration from aqueous-handling pump
j	Aspiration from organic-handling pump

**Table 4:** Sequence of steps for autonomous isolation of ^211^At from cyclotron bombarded Bi metal targets.

Step #	Step ID	Description	Step elapsed time, min (approx.)
1	Dissolution^a,b^	Dissolve Bi target in HNO_3_	75–90
2	Distillation^c^	Distill away HNO_3_, leaving Bi salts	
3	Bi salt conversion^d^	Dissolve/mix Bi oxynitrate salts in HCl	20
4	^211^At load 1	Extraction of ^211^At into DIPE, pass 1	14–17
5	^211^At load 2^e^	Extraction of ^211^At into DIPE, pass 2	
6	Wash 1	DIPE wash w/HCl	14
7	Wash 2	DIPE wash w/HCl	
8	Wash 3	DIPE wash w/HCl	
9	^211^At product collection	Dispense isolated ^211^At/DIPE to tube	1

^a^Bi target dissolution system and process described by O’Hara *et al*.^38^.

^b^Dissolved Bi target effluents are routed directly to pre-heated distillation chamber.

^c^Distillation time is dependent on mass of Bi metal in target.

^d^Prepared target solution is directly retrievable by solvent extraction system via distillation flask.

^e^Prior to the 2nd load step, the Bi-bearing aqueous phase is dispensed to/withdrawn from the distillation chamber to collect additional ^211^At from residual load solution residue.

**Table 5:** Reagent injections during the sequence of steps described in Table 4.

Step #	Reagent	Volume, mL	Footnotes
1	10 M HNO_3_	16	
2	—	—	
3	8 M HCl	17.5	a
4	Sol’n from #3	—	
	DIPE	7.6	b
5	—	—	
6	8 M HCl	9.5	c
7	8 M HCl	9.5	c
8	8 M HCl	9.5	c
9	—	—	

^a^HCl-dissolved Bi-bearing salts were observed to increase in volume to between ~19 and ~22 mL.

^b^DIPE was initially equilibrated with 8 M HCl.

^c^8 M HCl wash solution was initially equilibrated with DIPE; DIPE volume decreases slightly with each HCl/DIPE wash cycle.

Step 1 is performed in a fixed time interval, while the other steps have varying time duration. Step 2 is performed until the salts are visually observed to be dry. This is primarily determined by observing the elapsed time between condensate droplets within a jacketed condenser positioned between the distillation and distillate chambers. Depending on the mass of Bi in the target assembly, the elapsed time of the combined target dissolution and nitric acid distillation was ~75–90 min (distillation performed at 180–190 °C). This automated acid distillation process takes longer than the manual distillation method (which requires ~30–45 min), since a temperature of 300 °C is used there^21^. The automated process cannot be performed at this high temperature, since severe Bi salt spatter begins to occur above ~200 °C. Salt spatter from acid boiling/bumping places much of the ^211^At-bearing Bi oxynitrate salt out of reach of the 8 M HCl used to subsequently dissolve the saltcake (per step 3), and therefore needs to be avoided.

Upon completion of the distillation step (per a prompt of the software by the operator), the Bi salt conversion (step 3) is initiated. First, the heating block is rapidly cooled (by remote gating of chiller fluid through the block) to 75 °C. During the block cooling interval, the program pauses until a 75 °C heating block temperature is reached. Next, the still-warm Bi oxynitrate saltcake is dissolved by cycled dispensation/aspiration of 8 M HCl from pump 1 to/from the distillation chamber. A number of these cycles is required ensure the saltcake is fully dissolved (and the number of cycles performed is calibrated to successfully dissolve a saltcake resulting from the heaviest possible Bi target). Prior to initiation of the first solvent extraction step (step 4), the program ensures that the heating block temperature has been reduced to at least 35 °C via a second block cooling interval.

At the initiation of step 4, pump 1 aspirates the HCl-dissolved ^211^At/Bi solution out of the distillation chamber and proceeds with the DIPE load step. During the solvent extraction process, the time to perform steps 4–5 varies slightly (14–17 min), depending on the volume of the dissolved Bi-bearing solution; a heavy Bi target solution, due to its larger volume, will require more time to pass through the PBS during the phase boundary determination cycles vs. a lighter target solution. Once the ^211^At-depleted Bi solution is sent to waste and the wash cycles begin (steps 6–8), the elapsed times are very similar, since the aqueous and organic volume changes at this point are minimal. The autonomous solvent extraction process (steps 4–9) ranged between 29 and 32 min. Overall, a complete processing run required ~2.1 to ~2.4 elapsed hours between initiation of the Bi target dissolution and dispensation of the isolated ^211^At product in DIPE. The autonomous solvent extraction operations represent only ~22% of the total ^211^At processing time.

### ^211^At activity quantification

All ^211^At activity levels were decay corrected to EOB. Direct measurements of ^211^At activity levels were obtained using a CRC-15R dose calibrator (Capintec, Inc., Ramsey, NJ). The instrument was calibrated for ^211^At by UW; a setting of 040 was employed for ^211^At-bearing solutions in polyethene vials. The setting was based on a cross-calibration to a high purity Ge (HPGe) detector (Ametek, Oak Ridge, TN) that had been calibrated against NIST-traceable gamma standards. It has been demonstrated that high Bi concentrations attenuate the weak ^211^At and progeny X-ray and gamma emissions, thus resulting in negatively biased activity measurements^21^. Therefore, UW-determined correction factors were employed for samples containing well-known Bi concentrations. Otherwise, ^211^At samples high in Bi were not reported by dose calibrator. Rather, liquid scintillation analysis (LSA) was employed.

LSA was performed on direct sample aliquots or serial dilutions of ^211^At-bearing samples, depending on ^211^At activity levels present in the sample. Typically, 20 µL aliquots of each fraction (direct or serial dilution) were withdrawn and added to 4 mL scintillation cocktail that was pre-dispensed into a 2-dram polyethylene scintillation vial. The prepared samples were typically allowed to decay through one or more half-lives prior to alpha decay measurement by LSA (TriCarb 1900CA, PerkinElmer). Only LS measurements that exhibited ≤3500 cps were used, in order to assure linear LS analyzer response. If the sample measurement exceeded this count rate, it was allowed additional time to decay and then recounted. The counting region between 40 and 2000 channels, with tSIE quench correction activated, assured integration of the ^211^At and ^211^Po alpha emissions (5.87 and 7.45 MeV, respectively). Since ^211^At branch decays to an alpha emission either via ^211^At directly, or through ^211^Po^6^, the sum of the two alpha peaks, adjusted to a 95% detection efficiency, yielded the activity of ^211^At in the sample. The LS detection efficiency was determined as the ratio of the count rate of the LS alpha counting region and the ^211^At disintegration rate reported by the cross-calibrated dose calibrator.

All system components and effluents that came in contact with ^211^At were sampled for ^211^At activity during or at the completion of each run. Low Bi-bearing samples were analyzed by dose calibrator and LSA. Values and uncertainties shown on individual run results are the mean ± 1s obtained between the two analytical instruments. When the average result over multiple ^211^At processing runs are reported, the uncertainty is the sample standard deviation (±1s) across that set of runs.

### Bismuth analysis

The total mass of Bi in the isolated DIPE phase was determined following analysis of each sample by ICP-OES after undergoing the sample preparation method shown in Table 6.

**Table 6:** Method for determination of Bi^3+^ in DIPE phase following solvent extraction system tests with 8 M HCl containing ~3 g dissolved Bi metal.

Step	Treatment	Notes
1	Isolation of DIPE phase from post-contacted solutions	Determine volume of recovered DIPE fraction.
2	Evaporation of DIPE	Performed in heating block. Results in small amount of non-volatile residue.
3	Aqua regia treatment	Destruction of organic residues.
4	Closed vessel microwave digestion^a^	Complete destruction of organic residues; complete solubilization of Bi^3+^.
5	Bi analysis by ICP-OES^b^	

^a^3 mL 2.5 M HNO_3_ + 0.8 mL 30% H_2_O_2_ was added to sample residue in HP500 digestion vessels. Sample digested in a MARS 5 digestion system (CEM, Stallings, NC); temperature held at 180 °C for 30 min.

^b^Dilution of sample into ~2% Optima grade HNO_3_ prior to analysis.

Resulting solutions were analyzed using an iCap 6500 Duo (Thermo Scientific, Waltham, MA) ICP-OES with analysis performed in axial mode. An eight-point calibration curve was prepared for the Bi analysis by gravimetric dilutions of a 1000 µg/mL certified Bi standard purchased from High Purity Standards (Charleston, SC). Calibration curves used for the Bi standard concentrations had regression coefficients ≥0.9997; wavelengths of 190.2341, 222.8203, and 223.0602 nm were used in the analysis^46^. Analytical results were typically reported as being within ±5% at 2σ.

## Supplementary information

Electronic supplementary information
