# Supplementary material for: Development of an autonomous solvent extraction system to isolate astatine-211 from dissolved cyclotron bombarded bismuth targets
Source: Sci Rep. 2019 Dec 30;9:20318. doi: 10.1038/s41598-019-56272-7 (PMC6937302; doi:10.1038/s41598-019-56272-7)
Supplement: Supplementary file 1 — Electronic supplementary information [file 41598_2019_56272_MOESM1_ESM.pdf]

Development of an autonomous solvent extraction system to isolate astatine-211 from dissolved cyclotron bombarded bismuth targets

Matthew J. O’Hara[1]*, Anthony J. Krzysko[1], Donald K. Hamlin[2], Yawen Li[2], Eric F. Dorman[2], D. Scott Wilbur[2]

1. Nuclear Sciences Division, Pacific Northwest National Laboratory, 902 Battelle Blvd., PO Box 999, Richland, WA 99352
2. Department of Radiation Oncology, University of Washington, 616 N.E. Northlake Place, PO Box 355016, Seattle, WA 98105

*Corresponding author: matthew.ohara@pnnl.gov ; 509-375-5579 (ph); 509-375-5684 (fax)

Supplementary Information

S3 Discussion

During our early studies, it was observed that the mass of Bi metal in the cyclotron target assemblies ranged between 3.5 and 6.5 g. The importance of monitoring the Bi mass distribution became apparent when it was observed that the mass of Bi significantly affects the final solution volume once the HNO_3-dissolved Bi metal was evaporated to dry salts and the salts were dissolved in 8 M HCl.

Figure S1 plots the fractional volume increase in the added 8 M HCl (between 10 mL and 20 mL) after dissolving between 3.0 and 5.3 g Bi metal in conc. HNO_3 and then drying the solution to obtain Bi oxynitrate salts. The relative volume changes are observed to increase as 1) the mass of Bi present in the saltcake increases, and 2) as the 8 M HCl dissolving solution volume decreases. The dependence of final solution volume changes on these two parameters is shown as a contour plot in Figure S2.

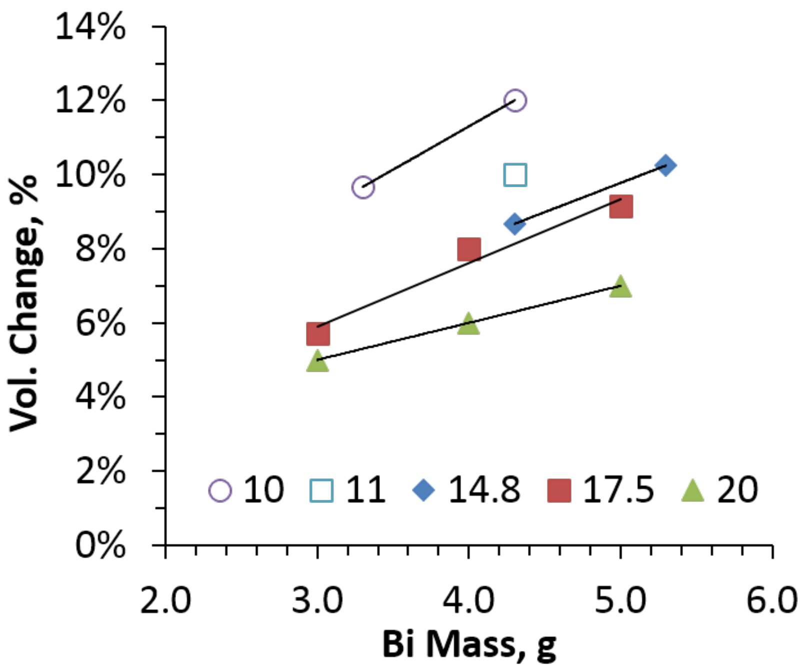

Figure S1. Percent volume change across a range of Bi metal masses after dissolution and conversion to Bi oxynitrate salts, followed by dissolution of the salts in 8 M HCl. Legend indicates the volume of 8 M HCl added to each saltcake.

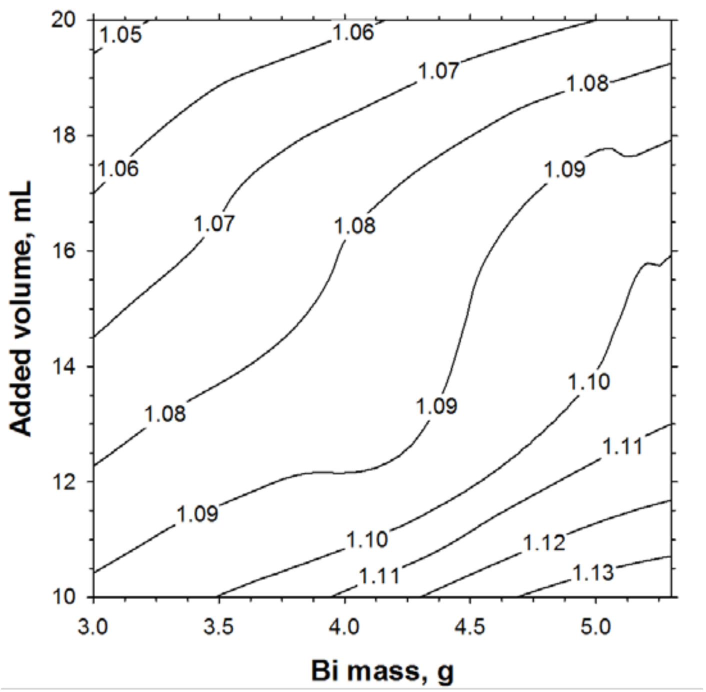

Figure S2. Fractional volume change of Bi^{3+}/HCl solution as a function of Bi metal mass dissolved and volume of 8 M HCl added.

S4.1 Manual method for ^{211}At isolation

The routine manual method for ^{211}At isolation from cyclotron bombarded Bi metal targets performed at the UW has been comprehensively described by Balkin et al.[11] A summary of the manual method is presented here.

Briefly, the Bi metal target is initially dissolved in a pool of conc. HNO_3. At the completion of the reaction, the dissolved target solution is poured into a distillation flask. The flask is connected to a chilled distillation head, and the flask is heated to ~300 °C in a heated block to distill away the nitric acid. The white salt residue is removed from the heating block and allowed to cool. Next, 10 mL of 8 M HCl is added to the salt residue; the solution is manually swirled in the flask until all Bi salts are dissolved. The solution is then added to a 20 mL glass LSC vial.

The prepared ^{211}At-bearing solution is then used in a solvent extraction process to isolate ^{211}At from the copious quantity of dissolved Bi salt. For convenience, a high-level summary of the solvent extraction portion of the method is provided in Table S1. DIPE is added to the LSC vial and mixed with a magnetic stir bar for 10 min (step 1). At the conclusion of the mixing interval, the vial is removed from the stirring block, the liquid mixture is allowed to gravity settle, and a transfer pipette is used to aspirate the aqueous (dense) phase and dispense it to waste.

Table S1. Manual method for ^{211}At isolation by solvent extraction. Organic extractant is 4.0 mL DIPE. [a]

Step	Description	Reagent add’n	Vol., mL	Mixing time, min	Stir rate, rpm
1	Extraction	Dissolved target [b]	10 [c]	10	800
2	Organic phase washes	8 M HCl [d]	5	5	800
3			5	5	800
4			5	5	800
5			5	5	800
6	Back-extraction	4 M NaOH	0.6 [e, f]	10	1000

a. DIPE is pre-contacted with 8 M HCl.
b. Bi target dissolved in HNO_3; solution evaporated to dry salt residue; salts dissolved in 8 M HCl.
c. Volume of 8 M HCl added; this does not reflect increased volume of final solution.
d. 8 M HCl is pre-contacted with DIPE to minimize DIPE solubility in aqueous phase.
e. Volume of 4 M NaOH added; this does not reflect increased volume of final ^{211}At product.
f. Subsequent additions of 4 M NaOH (and further mixing) may be required if ^{211}At product solution has a pH ≤13.

Next, four washes of the organic phase with 8 M HCl are performed sequentially, with five minutes of stirring time each. The wash solutions are discarded to waste by a transfer pipette. Finally, ^{211}At in the acid-washed DIPE is back-extracted into a small volume of 4 M NaOH. After mixing, the aqueous phase, which contains the isolated ^{211}At, is withdrawn. The elapsed time to execute the solvent extraction procedure is ~50-55 min.

S4.5 Engineered solvent extraction system and sample processing

Figure S3 shows an image of the autonomous solvent extraction system that is illustrated in Figure 7. The labeled components are described in Table S2.

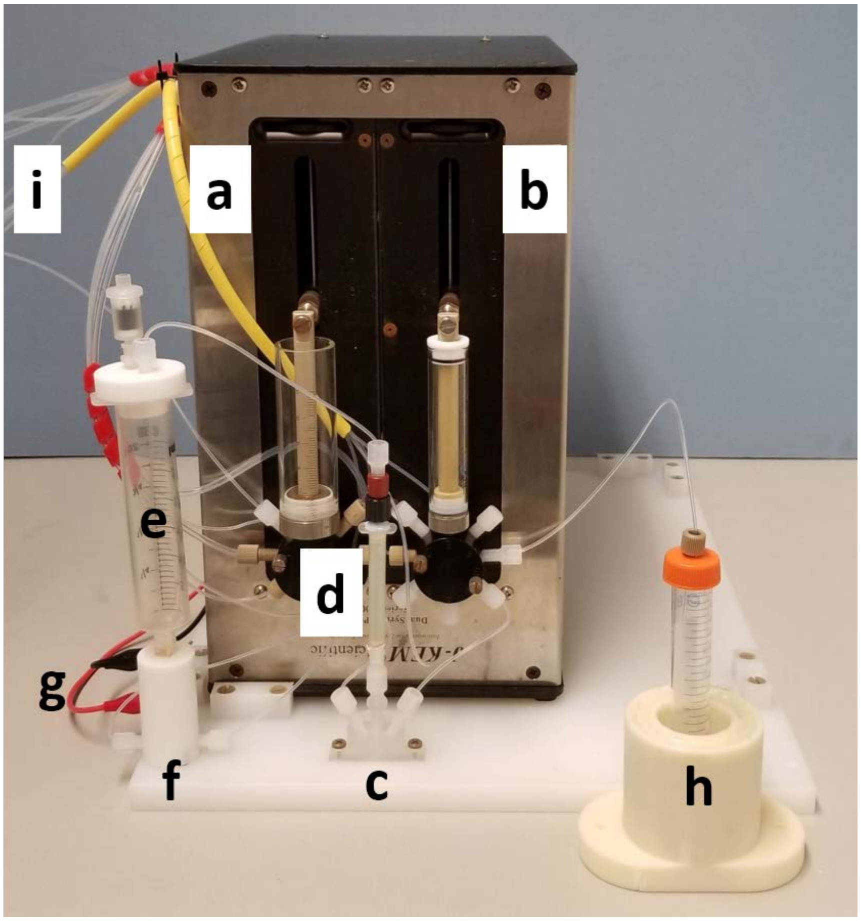

Figure S3. Front face of the two-pump solvent extraction system for ^{211}At isolation from dissolved Bi target. Table S2 identifies the labels. The reagent and waste bottles (not shown) are located to the left of the pump boxes.

Table S2. Identification of solvent extraction system labels shown in Figure S3.

Label	Component	Notes
a	Pump 1 (aqueous)	25 mL syringe; 8 port distribution valve
b	Pump 2 (organic)	10 mL syringe; 8 port distribution valve
c	Mixing tee	Aqueous / organic fluids enter tee from each pump
d	Column mixer	Liquids exiting (c) pass through the column mixer to form a brief emulsion
e	Phase settling reservoir (PSR)	Aqueous / organic phases from (d) collect in the PSR and are allowed to gravity settle
f	Phase boundary sensor (PBS)	Sensor determines when all aqueous phase has been withdrawn from (e)
g	Electrodes	Connecting wires placed between the coiled Pt wires embedded with the PBS and the syringe pump I/O board
h	^{211}At product collection vial	Isolated ^{211}At in DIPE
i	Reagent in / waste out lines	

References

[1] E.R. Balkin, D.K. Hamlin, et al., Evaluation of a wet chemistry method for isolation of cyclotron produced [^{211}At]astatine, Applied Sci, 3 (2013) 636-655.

Acknowledgements

This research is supported by the U.S. Department of Energy Isotope Program, managed by the Office of Science for Nuclear Physics. The Pacific Northwest National Laboratory is operated for the U.S. Department of Energy by Battelle under contract DE-AC06-67RLO 1830.
